# Energy-Expenditure Estimation During Aerobic Training Sessions for Badminton Players

**DOI:** 10.3390/s25196257

**Published:** 2025-10-09

**Authors:** Xinke Yan, Jingmin Yang, Jin Dai, Kuan Tao

**Affiliations:** 1 Sports Coaching College, Beijing Sport University, Beijing 100084, China; 2024210065@bsu.edu.cn (X.Y.); daijin@bsu.edu.cn (J.D.); 2 School of Sports Engineering, Beijing Sport University, Beijing 100084, China; yangjingmin@bsu.edu.cn

**Keywords:** badminton, energy expenditure, wearable sensors, machine learning, personalized training

## Abstract

This study investigated differences in energy-expenditure (EE) modeling between badminton players of varying competitive levels during aerobic training. It evaluated the impact of sensor quantity and sample size on prediction model accuracy and generalizability, providing evidence for personalized training-load monitoring. Fifty badminton players (25 elite, 25 enthusiasts) performed treadmill running, cycling, rope skipping, and stair walking. Data were collected using accelerometers (waist, wrists, ankles), a heart rate monitor, and indirect calorimetry (criterion EE). Multiple machine learning models (Linear Regression, Bayesian Ridge Regression, Random Forest, Gradient Boosting) were employed to develop EE prediction models. Performance was assessed using R^2^, mean absolute percentage error (MAPE), and root mean square error (RMSE), with further evaluation via the Triple-E framework (*Effectiveness*, *Efficiency*, *Extension*). Elite athletes demonstrated stable, coordinated movement patterns, achieving the best values for R^2^ and the smallest errors using minimal core sensors (typically dominant side). Enthusiasts required multi-site sensors to compensate for greater execution variability. Increasing sensors beyond three yielded no performance gains; optimal configurations involved 2–3 core accelerometers combined with heart rate data. Expanding sample size significantly enhanced model stability and generalizability (e.g., running task R^2^ increased from 0.49 (*N* = 20) to 0.95 (*N* = 40)). Triple-E evaluation indicated that strategic sensor minimization coupled with sufficient sample size maximized predictive performance while reducing computational cost and deployment burden. Competitive level significantly influences EE modeling requirements. Elite athletes are suited to a “low-sensor, small-sample” scenario, whereas enthusiasts necessitate a “multi-sensor, large-sample” strategy.

## 1. Introduction

Badminton is a dynamic and highly technical sport, which requires rapid responses and transitions for serve, stroke, and racquet- and ball-handling skills [1]. Faster ball speed, greater swing frequency, larger hitting strength, and shorter interval time all concern sudden acceleration or deceleration, abrupt changes in direction, and jumps [2,3] for players to compete effectively, setting a stringent standard of agility, speed, upper- and lower-limb strength, and coordination [4]. Well-trained players adaptively reallocated physical demands on both aerobic and anaerobic systems during multi-round plays [5], with 60–70% of energy derived from aerobic and 30% from anaerobic systems [1,6,7]. The sufficient aerobic capacity supports the recovery from intermittent anaerobic exercises [8], reducing fatigue [9] and injuries [10] and elevating performance.

Therefore, proper training sessions should be oriented towards aerobic exercises for badminton players. Standard laboratory-based training types include treadmill exercise [11] or power bike cycling [1], which aim at testing endurance and speed. Otherwise, rope skipping [12] and stair climbing [13] are adopted to gauge muscle strength, agility, and body coordination as well. The acute effect of measuring the intensity of aerobic training is to calculate energy expenditure (EE) [4,14]. Over the past decades, EE has been measured with relative accuracy through direct calorimetry [15], indirect calorimetry [16], and the doubly labeled water (DLW) method [17]. However, these approaches are limited by their inability to quantitatively determine the intensity of training, as well as by large operational costs and constraints in capturing EE for specific types of physical activity [18]. Thus, the pursuit of indirect measurement of EE remains a challenge.

Compared with traditional methods, the rapid development of modern wearable technology has offered a more practical approach to estimating EE. By integrating sensors such as accelerometers, heart rate monitors [19], and the SenseWear Armband [20] and by leveraging machine learning [21] or deep learning techniques [22], these methods provide low-cost, portable solutions that are well-suited for continuous monitoring in both free-living conditions and diverse physical-activity tasks. However, such methods also present certain limitations. Sensor signals are affected by wearing position [23], exercise modality [24], and individual variability [25], meaning that different physical tasks may require distinct sensor deployment strategies. On the other hand, although increasing the number of sensors can enhance data dimensionality, redundant signals may reduce model performance while leading users to feel uncomfortable and increasing computational cost. Moreover, these models often lack sufficient generalization, with errors frequently doubling when applied to new individuals who were not pretrained [26,27], making it difficult to adapt them to diverse populations or scenarios.

Meanwhile, individual differences in exercise energy-expenditure prediction may not only manifest in physiological signals [25] but also in sensor signals such as acceleration, which can vary depending on the athlete’s performance level. Existing studies on badminton strokes have shown that athletes of different skill levels exhibit significant differences in movement patterns, action structures, limb acceleration distributions, and bodily coordination [28,29,30]. Specifically, elite athletes demonstrate superior upper- and lower-limb coordination and more efficient center-of-mass transfers during strokes [28], while amateur participants tend to exhibit greater individual variability and instability in movement execution. These differences imply that the energy output information embedded within sensor signals may vary depending on the athlete’s performance level. Since energy-expenditure prediction models rely on the quality and structural consistency of kinematic data, such as acceleration, these inter-level variations may directly affect model accuracy and generalization. Therefore, athletes of different performance levels may require distinct input feature configurations and exhibit differentiated modeling characteristics in EE prediction, rather than being modeled under a single unified standard.

Therefore, we need to explore what factors affect sensor signals, how these influences ultimately impact the energy-consumption prediction model for badminton aerobic training, and simultaneously find suitable sensor simplification strategies to balance accuracy and cost. To address this, we adopted and extended the Triple-E principle framework [31] to comprehensively assess the model’s performance in energy consumption prediction. In our previous work, we introduced and explained the concept of economical equilibrium embodied in this framework; building upon that foundation, the present study incorporated sample size and sensor count as key evaluation factors to investigate appropriate sensor simplification strategies during different aerobic exercise modalities.

This study derived a modeling strategy for energy expenditure for badminton players of different skill levels and investigated how sample size and sensor configuration affected modeling accuracy and efficiency. Through this research, we aimed to provide more personalized energy-expenditure prediction models for the badminton training field, offering scientific evidence for athlete-training load management and recovery strategies.

## 2. Materials and Methods

### 2.1. Participants

A total of 50 right-handed badminton athletes from Beijing Sport University participated in this study, comprising 25 elites and 25 enthusiasts (see Table 1). Athletes in the elite group met the inclusion criteria of holding a National Second-Class (or higher) sports classification and engaging in at least two badminton training sessions per week. In contrast, enthusiasts had less than two years of formal badminton training and no specialized background in track and field, cycling, rope skipping, or climbing. Participants who had musculoskeletal injuries such as fractures, sprains, or tendon damage within six months prior to the study were excluded from this research. Prior to testing, participants were instructed to abstain from strenuous exercise and caffeine intake, and to ensure they were free from cold or fever symptoms. Female participants were excluded from testing during their menstrual period. None of the participants had taken any medication prior to the experiment. Informed consent was obtained from all participants, and demographic data, including age, height, body mass, and years of training experience, were collected for analysis.

The study protocol was approved by the Ethics Committee of Beijing Sport University (Approval No. 2025079H). All experiments were conducted in a controlled laboratory environment at the School of Sports Engineering, Beijing Sport University, with constant temperature and humidity maintained throughout the testing sessions.

### 2.2. Procedure

Prior to the experiment, participants were instructed on the use of the equipment, and body composition data, including body mass, fat mass, muscle mass, and body fat percentage were collected using a body composition analyzer. Height was measured using a standardized stadiometer with a precision of 0.1 cm.

**Figure 1:**
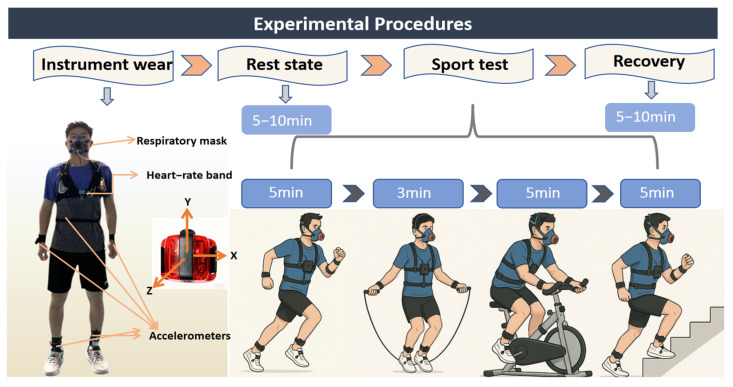
Experimental procedures.

Each participant was equipped with a portable indirect calorimetry system (Metamax 3B, Cortex Biophysik, Leipzig, Germany) alongside a Polar heart rate monitor (Polar H7, Kempele, Finland) to record their heart rate on a per-minute basis. The heart rate data were synchronized via Bluetooth with the Metamax 3B system. Participants wore accelerometers (ActiGraph GT3X+, Pensacola, FL, USA), a research-grade device widely used to assess physical activity levels in both healthy and clinical populations [32,33,34,35,36,37], on the waist, both ankles, and both wrists (see Figure 1), and movement data were recorded at a sampling rate of 60 Hz.

Following the secure placement of all devices, participants were instructed to remain still until physiological parameters stabilized. Baseline resting metabolic data were collected during a 5–10 min pre-exercise period. Participants received an activity checklist with descriptions at the start of testing, and the activity order was rotated every 4–5 participants to minimize order effects [38]. The exercise protocol comprised four distinct aerobic activities: treadmill running (Mercury 4.0 training treadmill) at 8–10 km/h for 5 min, cycling (Ergoline Ergoselect 100K, Bitz, Germany) at 120–150w and 60–70 rpm for 5 min, rope skipping (Li Ning brand) with two-foot single-unders for 3 min, and stair walking (30 cm training step) at 60 steps per minute for 5 min (refer to Figure 1); participants self-selected exercise intensities within the prescribed ranges. Rest intervals between exercises were determined based on heart rate recovery, followed by a 5–10 min cool-down period after completing all exercise tests.

### 2.3. Statistical Analysis

Five categories of data were collected: respiratory gases (the gold standard for measuring energy expenditure), heart rate (HR) and ΔHR (the difference between activity and resting heart rates), raw accelerometer data, demographic information, and body composition. Respiratory gas data were stored in memory and downloaded using MetaMax Soft Studio ActiLife version 6.9; according to the ActiGraph guidelines, accelerometer data from all five positions were downloaded, including *x*-, *y*-, *z*-axis and vector magnitude (VM) counts, then exported to Microsoft Excel for averaging. To ensure accuracy, redundant segments were removed based on time markers during the test, and steady-state data were selected by extracting the penultimate minute of each activity. All accelerometer counts and raw data were processed in the same manner as the gas exchange data to ensure consistency. For each participant, demographic, anthropometric, accelerometry, and physiological features were extracted, including age, sex, height, body mass, BMI, and fat mass. Basic participant information and experimental data were summarized using means ± standard deviations (*x* ± *s*). (In this study, due to unstable Bluetooth connections, several participants experienced data loss during different training tasks; to ensure the rigor of the experiment, data from 40 participants were ultimately selected for analysis). The collected features were used as inputs for machine learning models, including linear models such as linear regression and Bayesian ridge regression; nonlinear models such as random forest regressor, gradient boosting regressor, support vector regressor (SVR), and XGboost regressor; as well as tree-based models like decision tree regressor. All possible combinations of features were generated and iteratively input into each model with default parameters to train and evaluate predictive models specific to the four aerobic exercise modalities, aiming to identify the most relevant features and the most accurate model for each activity. Model performance was assessed using mean absolute percentage error (MAPE), R-squared (R^2^), and root mean square error (RMSE), calculated using Python 3.13.0 and validated through five-fold cross-validation to ensure the robustness and generalizability of the predictive outcomes. The best model is defined as the one with the best R^2^ and the smallest errors (MAPE, RMSE).

### 2.4. Triple-E Evaluation Framework

Building upon the Triple-E framework, we quantified specific metrics to evaluate and score each model and dataset combination, thereby facilitating an optimal balance between accuracy and practicality.

*Effectiveness* = *R*^2^ × (1 − *MAPE*) (1)

This metric assesses the predictive precision of the model; higher values indicate superior accuracy.

*Efficiency* = 1 − (*X*/*Y*) − *α* (2)

Let *X* be the number of sensors, *Y* the maximum number of sensors, and *α* the model complexity penalty values. The model complexity penalty values were assigned as follows, with 0 in linear regression and Bayesian ridge models; 0.1 in decision tree and SVR models; and 0.2 in random forest, XGBoost, and gradient boosting models. This metric rewards models that use fewer sensors and simpler algorithms, reflecting practical deployment considerations.

(3) $$\mathrm{Extension}=[N/N_{\mathrm{ref}}\cdot R^{2}]/[1+(N/N_{\mathrm{ref}}\;-\;1)\cdot R^{2}]$$

To evaluate the impact of different sample sizes on the accuracy and stability of the estimation, we denoted the sample size used in this study as *N* and referred to the commonly reported sample size in the literature as *N*_*ref*_. (In this study *N* = 20, *N*_*ref*_ = 40).

## 3. Results

### 3.1. Differences in Key Sensor Locations Between Skill Levels in Badminton Athletes

Model performance across different exercise types and badminton players of different skill levels was evaluated using R-squared (R^2^), mean absolute percentage error (MAPE), and root mean square error (RMSE). The results are shown in Table 2. For running, the best-performing model in the elite group was the gradient boosting regressor, with input features including height, BMI, body fat percentage, skeletal muscle, gender, right wrist VM, and waist VM. In the enthusiast group, the best-performing model was the random forest regressor, with input features including skeletal muscle, resting heart rate, and VM signals from five positions (left wrist, right wrist, left ankle, right ankle, and waist).

In rope-skipping training, the optimal model for the elite group was linear regression, which took height, right wrist VM, right ankle VM, and ΔHR as inputs, whereas the random forest regressor performed best in the enthusiast group using height, body mass, gender, left wrist VM, and waist VM as inputs.

For cycling, the elite group’s optimal model was the random forest regressor with input variables including body fat percentage, gender, right wrist VM, right ankle VM, ΔHR, HR, and resting heart rate, while the Bayesian ridge regression model performed best in the enthusiast group with features including height, BMI, skeletal muscle, gender, left ankle VM, right wrist VM, right ankle VM, waist VM, and resting heart rate.

In the stair walking task, the gradient boosting regressor using height, body mass, BMI, body fat percentage, left wrist VM, right wrist VM, HR, and resting heart rate as inputs provided the best results for the elite group, whereas in the enthusiast group, the linear regression model achieved the greatest R^2^ using body mass, skeletal muscle, gender, right wrist VM, and ΔHR as inputs.

### 3.2. Impact of Sample Size and Number of Sensors on Aerobic Energy-Expenditure Modeling Accuracy in Badminton Athletes

#### 3.2.1. Effect of Sensor Quantity

With the sample size held constant at *N* = 20 participants, each equipped with six sensors (one heart rate strap and five accelerometers), and under identical model structures and training parameters, predictive models were constructed for the four aerobic training tasks to evaluate the influence of sensor quantity on model performance.

**Figure 2:**
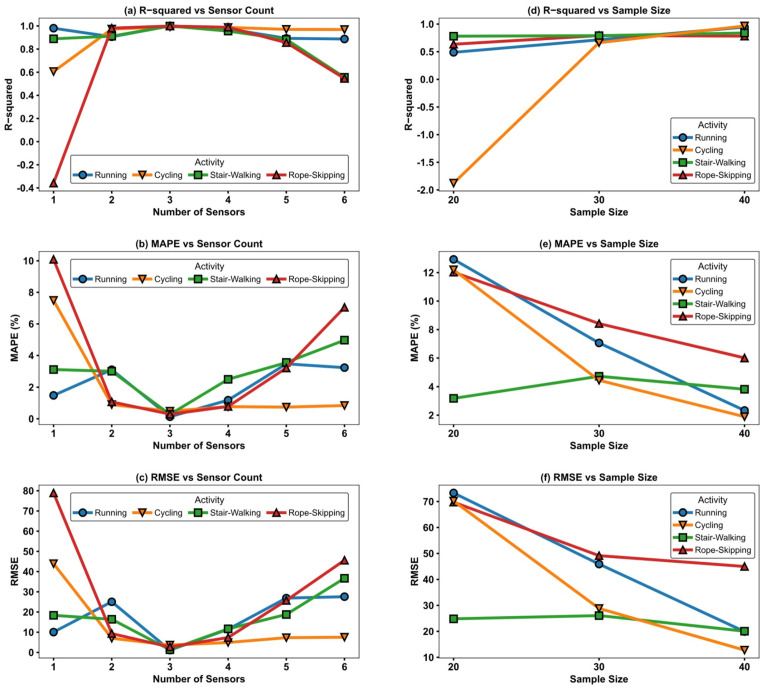
Model error evaluation for different sample sizes and different sensor counts. (**a**–**c**) Model error evaluation across varying numbers of sensors. (**d**–**f**) Model error evaluation across different sample sizes.

The results (see Figure 2) indicated that increasing the number of sensors and information dimensions did not continuously enhance model performance. On the contrary, the best R^2^ and smallest error were achieved when using three sensors, specifically, two accelerometers at the optimal body locations combined with heart rate data, while further increasing the number of sensors to five or six led to a notable decline in model performance. For instance, during the rope-skipping task, the two-sensor configuration yielded an R^2^ of 0.981 and a MAPE of 1.07%, indicating a tightly controlled prediction error; when expanded to a three-sensor setup, the R^2^ increased to 0.998 and the MAPE dropped to 0.29%, representing the optimal configuration. However, using five or six sensors led to increased prediction errors, with the R^2^ and MAPE dropping to 0.855 and 0.545, respectively. 

#### 3.2.2. Effect of Sample Size

To systematically investigate the role of sample size in the construction of energy-expenditure prediction models, this study established training datasets with sample sizes of 20, 30, and 40 participants, respectively (using only heart rate sensor data), and developed and evaluated models under identical model architectures, training parameters, and feature conditions. For each exercise, the model and feature combination that achieved the best R-squared when using the full dataset of 40 participants was selected for comparison across different sample sizes. Results (Figure 2) demonstrated a steady improvement in both the stability and accuracy of predictions as sample size increased. Taking treadmill running as an example, increasing the sample size from 20 to 40 participants improved the model’s R^2^ from 0.49 to 0.95, while the MAPE decreased from 12.93% to 2.33%, and the RMSE was correspondingly reduced. The model’s ability to fit EE was significantly enhanced. Notably, in cycling tasks with a smaller sample size (20 participants), heterogeneity in movement patterns, physiological responses, and energy expenditure disrupted the model performance, resulting in negative R^2^ values on the test set.

### 3.3. Comprehensive Evaluation of Aerobic Energy-Expenditure Prediction Models in Badminton Based on the Triple-E Framework

Based on the above results and the proposed Triple-E framework, we selected three representative configurations of sample size and sensor count and calculated the area of the two-dimensional triangle formed by their coordinates within the metric-defined space, where a larger area indicated more balanced or stronger overall performance across the three dimensions.

The first configuration was chosen based on the best R^2^ sensor setup identified in Figure 2 (*N* = 20, Sensors = 3). The second configuration (*N* = 20, Sensors = 2) included a heart rate strap and one accelerometer positioned at the right wrist, as the vector magnitude (VM) at this site was consistently important across all training tasks (Table 2), and this two-sensor setup demonstrated only a marginally smaller R^2^ than the three-sensor configuration. The final configuration involved the largest sample size with a minimal sensor setup (*N* = 40, Sensors = 1), utilizing only heart rate data.

**Figure 3:**
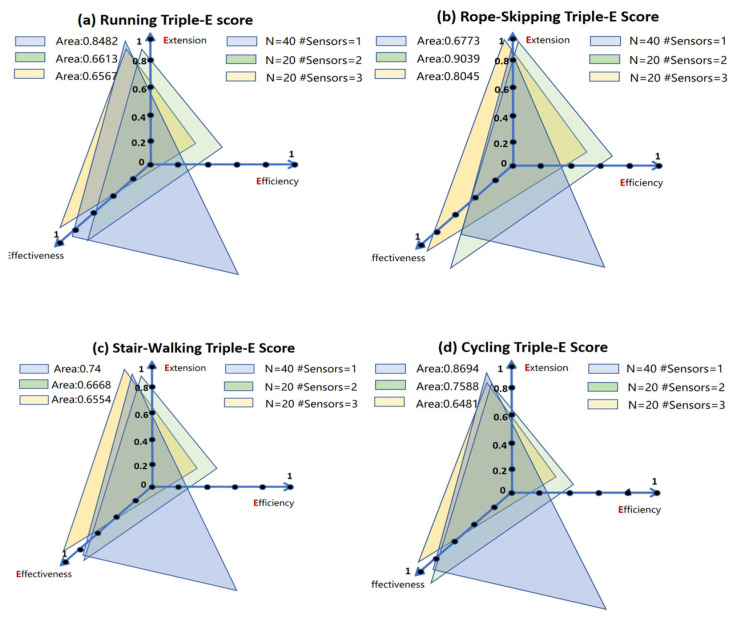
Triple-E score for four kinds of aerobic training. Conversely, in rope-skipping training (Figure 3), the *N* = 20, Sensor = 2 configuration yielded the best composite performance (Area = 0.9039), achieving greater *Efficiency* scores relative to running, attributed to the reduced computational burden from the simplified model. The *N* = 40, Sensor = 1 group showed a notably smaller area (0.6773), primarily due to substantial losses in *Effectiveness*.

Taking running and rope-skipping training as examples, in running training (Figure 3), different configurations were evaluated in terms of *Effectiveness*, *Efficiency*, and *Extension* using Equations (1), (2), and (3), respectively. It was found that there were significant differences in the score distributions across the three dimensions. The N = 40, Sensor = 1 group achieved the best overall performance, with the largest triangular area (Area = 0.8482), demonstrating marked advantages in the *Extension* and *Efficiency* dimensions while incurring only a slight reduction in *Effectiveness*. Although the Sensor = 3 and Sensor = 2 groups performed reasonably well in *Extension* and *Effectiveness*, their lower *Efficiency* scores resulted in smaller overall areas (Area = 0.6567 and 0.6613, respectively). The training conditions of stair walking and cycling were similar to those of running training.

## 4. Discussion

This study aimed to address the challenges in energy-expenditure monitoring for badminton players, particularly in optimizing the configuration of wearable sensors and machine learning models. While traditional methods were accurate, they were difficult to apply widely due to expensive costs and operational complexity. In contrast, wearable sensors offered a more economical alternative, but they faced challenges such as signal interference and individual variability. This research innovatively considered skill level differences and proposed a personalized energy-expenditure prediction model. By optimizing sensor quantity, placement, and sample size, the model’s accuracy and generalizability were improved. The study also provided coaches with guidance based on the Triple-E framework, helping them choose the most appropriate monitoring strategy and balance accuracy and efficiency across different training tasks and skill levels, enabling the development of personalized training and recovery plans. By analyzing the modeling features of the elite and enthusiast groups, this study reveals that EE prediction models for elite badminton athletes typically rely on a smaller number of sensors, concentrated on the dominant right-side limb, particularly the right wrist accelerometer (see Table 2). This simplified feature configuration may be related to the distinctly different upper-limb muscle activation and movement coordination patterns between elite and lower-level athletes in specific tasks [39], with elites demonstrating more stable and repeatable movement structures in training [28]. In contrast, badminton enthusiasts tended to require input from multiple sensors, such as those on the wrist, ankle, and waist, to reach acceptable prediction performance. This suggests greater individual variability and bodily fluctuation in their movements, with less uniformity, forcing the model to depend on more diverse information sources to improve the interpretation of EE patterns. An exception was found in the stair-walking task, where the optimal accelerometer placement for enthusiasts was also the right wrist alone. This may be explained by the relatively gentle prescribed intensity of stair climbing (minimum mean heart rate among the four exercises) and the randomized exercise order; when stair walking occurred first, enthusiasts and elite athletes likely performed similarly. Excluding this special case, our findings were consistent with the prior literature: experienced, high-level athletes exhibit superior body control and efficient movement patterns, with more precise and stable force applications [40,41,42]. These findings provide a basis for future work on optimizing sensor deployment and personalizing training-load monitoring.

In this study, we systematically evaluated the dual impact of sensor quantity and sample size on model performance, finding that both factors were influential for modeling accuracy and robustness. Regarding sensor quantity, when the sample size of elite athletes was fixed (*N* = 20), model performance did not increase linearly with the number of sensors. Instead, optimal models most often occurred when only two to three key sensors were used, typically including core locations such as the right wrist accelerometer and a heart rate strap. For example, in rope-skipping tasks, the best prediction performance was achieved using three sensors (right wrist, right ankle, and heart rate). Adding non-core sensors beyond this configuration led to a decline in performance, likely due to redundant or noisy signals that interfered with feature extraction and generalization. This finding suggests that “more sensors” is not necessarily better, and the selective inclusion of core-location signals is essential, as core sensors can deliver excellent predictive accuracy [23,43,44]. For sample size, results showed that increasing the number of participants (from 20 to 40) improved model fit and stability. In summary, an effective sensor simplification strategy should prioritize core sensor locations, while increasing sample size remains decisive for boosting model accuracy.

After introducing the Triple-E (*Effectiveness*, *Efficiency*, *Extension*) framework, we conducted a systematic multidimensional evaluation of different sensor configurations and sample-size schemes. Our analysis revealed that in running, stair-walking, and cycling tasks, larger sample sizes were generally associated with greater Triple-E scores, reflecting a strong generalization and increased efficiency, particularly suitable for large-scale deployment or field applications. However, in rope skipping, the greatest score was achieved with Sensor = 2, suggesting that for high-variability activities, retaining a modest number of key sensors is more critical, as sample size alone could not fully compensate for insufficient feature representation. Moreover, a comparison between Sensor = 2 and Sensor = 3 configurations across all activities showed that, for elite athletes, a single accelerometer on a core body location yielded better balance across the Triple-E dimensions than using two accelerometers on core locations, even though the latter slightly improved accuracy. These findings indicate that model design should be flexibly adjusted according to task complexity, target population level, and wearable deployment scenarios, while acknowledging that a trade-off between accuracy and practicality is inevitable [45].

These findings are not only theoretically significant but also provide practical guidance for implementing training programs. For elite athletes, adopting an efficient configuration with a minimal number of core sensors, such as a right wrist accelerometer with a heart rate strap, is recommended. This setup reduces wearing burden and data processing costs and enables streamlined monitoring of training load. For enthusiasts and individuals with inconsistent movements, a strategy using multiple sensors and a larger sample size is recommended. This approach improves the model’s adaptability to movement fluctuations and provides more precise feedback. As skill levels improve, gradually shifting to simplified configurations helps balance comfort and accuracy. Coaches can use the Triple-E framework as a decision-making tool that considers predictive accuracy, deployment cost and contextual adaptability. Its composite score can optimize training prescriptions and tailor training loads for athletes at different performance levels, enhancing both personalization and the scalability of monitoring.

This study’s results demonstrate potential applicability across diverse populations. The approach may be extended to elite athletes in other sports, youth athletes, rehabilitation patients, and older adults, providing a foundation for individualized exercise monitoring and energy-expenditure assessment. The model may also be applicable to left-handed athletes, although differences in dominant-limb use and movement symmetry compared with right-handed athletes should be considered. Future modeling efforts could include bilateral sensor-based feature selection to allow automatic identification of the dominant limb and maintain predictive accuracy across both left- and right-dominant populations. This would improve the model’s generalizability and practical value.

Several limitations must also be acknowledged. The experiments were conducted under controlled laboratory conditions where exercise intensity, duration, and task order were strictly regulated. Consequently, the findings may not fully represent the diversity of outdoor or self-paced training scenarios. Outdoor running and cycling may be influenced by terrain slope and ground type [46,47], wind resistance, and ambient temperature, which can alter energy-expenditure patterns compared with indoor data. In addition, real training sessions often involve self-selected pacing, intermittent pauses, or brief sprints, which can introduce noise into sensor signals and reduce model stability. Long-duration wear presents additional challenges, as sensors may shift due to sweat, strap loosening or vigorous movement, leading to data loss or signal drift. Future research should collect data in real-world or mixed environments over extended periods, integrate terrain and weather information, and systematically evaluate model robustness under natural training conditions. Future investigations should also examine the effects of sensor-wear compliance, data-loss compensation, and environmental factors on predictive performance.

## 5. Conclusions

This study’s results revealed significant differences in the modeling characteristics of energy-expenditure prediction during aerobic training among athletes at different competitive levels. For badminton training-load monitoring, strategies should be tailored to skill levels; elite athletes can effectively detect training loads using sensor signals from core areas like the right wrist, while enthusiasts require multiple sites sensor signals to achieve excellent prediction accuracy. Analysis based on the Triple-E framework shows that sensor combinations should vary with training tasks. For routine exercises such as running and cycling, using a few or even a single sensor balances efficiency and cost, whereas complex movements like rope skipping demand sufficient core areas sensor signals to ensure accuracy. These findings provide a quantitative foundation for badminton training-load monitoring, injury prevention, and personalized training programs.

## Figures and Tables

**Table 1:** Basic information of the participants.

	Elite	Enthusiast
Participants (*N*)	25	25
Age (years)	21.1 ± 1.8	22.4 ± 2.2
Height (cm)	174.1 ± 6.3	172.8 ± 6.6
Body mass (kg)	70.8 ± 9.6	65.3 ± 6.8
BMI (kg/m^2^)	23.2 ± 2.1	21.9 ± 1.5
Fat (%)	17.3 ± 7.7	16.5 ± 8.8
Muscle (kg)	33.2 ± 5.9	30.9 ± 6.0

**Table 2:** Model Performance Summary by Group.

Group	Metric	Stair	Cycling	Rope Skipping	Running
Elite	Model	Gradient Boosting Regressor	Random Forest Regressor	Linear Regression	Gradient Boosting Regressor
Elite	Best accelerometer Combination	Left wrist VM, Right wrist VM	Right wrist VM, Right ankle VM	Right wrist VM, Right ankle VM	Right wrist VM Waist VM
Elite	R-squared	0.9994	0.9957	0.9984	0.9997
Elite	MAPE (%)	0.2784	0.4136	0.2916	0.1264
Elite	RMSE	1.3208	2.7995	2.6902	1.1323
Enthusiast	Model	Linear Regression	Bayesian Ridge	Random Forest Regressor	Random Forest Regressor
Enthusiast	Best accelerometer Combination	Right wrist VM	Left ankle VM, Right wrist VM, Right ankle VM, Waist VM	Left wrist VM, Waist VM	Left wrist VM, Left ankle VM, Right wrist VM, Right ankle VM, Waist VM
Enthusiast	R-squared	0.9998	0.9993	0.9969	0.9997
Enthusiast	MAPE (%)	0.1895	0.1444	0.6152	0.0608
Enthusiast	RMSE	1.1653	0.8465	3.5417	0.385

## Data Availability

The data supporting this study’s findings can be obtained from the corresponding authors upon reasonable request.
